# Management of Popliteal Pseudoaneurysm Associated With a Baker’s Cyst

**DOI:** 10.7759/cureus.112898

**Published:** 2026-07-18

**Authors:** Martin Safari Aketch, Zeeshan Ahmed

**Affiliations:** 1 Urology, Royal College of Surgeons in Ireland, Dublin, IRL; 2 Vascular Surgery, University Hospital Limerick, Limerick, IRL

**Keywords:** baker's cyst, bovine pericardial patch, popliteal artery pseudoaneurysm, vascular reconstruction, vascular surgeon

## Abstract

A 60-year-old male patient presented to the vascular clinic with a three-month history of a large popliteal fossa swelling post Baker's Cyst Aspiration. This swelling had been getting progressively larger over this period. CT angiogram of his lower limb reported a right popliteal aneurysm of 8.5 cm diameter with a large amount of intramural thrombus that was later confirmed as a pseudoaneurysm in our vascular multi-discliplinary meeting. It was decided to dissect the popliteal artery from a posterior approach and perform a bypass using a vein. Intraoperatively, a biological bovine pericardial patch was used for repair of his pseudoaneurysm. Postoperatively the patient was well from both a surgical point of view and suffered mild symptoms of COVID-19. Open surgery with primary repair with bovine pericardial patch demonstrated an alternative to treat this condition.

## Introduction

Pseudoaneurysms of peripherals arteries are not uncommon. They occur when a breach in the arterial wall can produce a contained hematoma formation, unlike a true aneurysm which involves dilation of all three vessel layers.

Pseudoaneurysms are most frequently seen in the common femoral artery, associated with IV drug abuse or after iatrogenic injury [1]. However, a pseudoaneurysm of the popliteal artery is rare, with penetrating injury or blunt trauma accounting for the majority of cases [2]. Other causes of this phenomenon include either knee procedures or exostosis [3]. The demographics tends to be young males similar to our case [3]. Kao and Chang's case also highlighted how rare this case is, with very little literature on popliteal pseudoaneurysms [4].

In its presentation, a popliteal pseudoaneurysm will present similar to a normal aneurysm as a pulsating mass, pain and nerve compression as the primary symptoms [5]. On the other hand, Raherinantenaina et al.'s case on popliteal pseudoaneurysm showed that the symptoms at presentation can be very subtle for a pseudoaneurysm and that minor trauma to the popliteal artery could result in a pseudoaneurysm [6].

## Case presentation

A 60-year-old male patient presented to the vascular clinic with a three-month history of progressively enlarging swelling involving the back of his right knee. He was unable to fully flex his knee. There were no typical intermittent claudication symptoms or any other stigmata of vascular disease. He reported that he had been treated for a Baker’s cyst previously by his family physician. He has a medical history of acute coronary syndrome, hypertension, and hypercholesterolemia. There was no family history of connective tissue disorders. He was a non-smoker and denied any history of intravenous or other recreational drug use.

**Figure 1:**
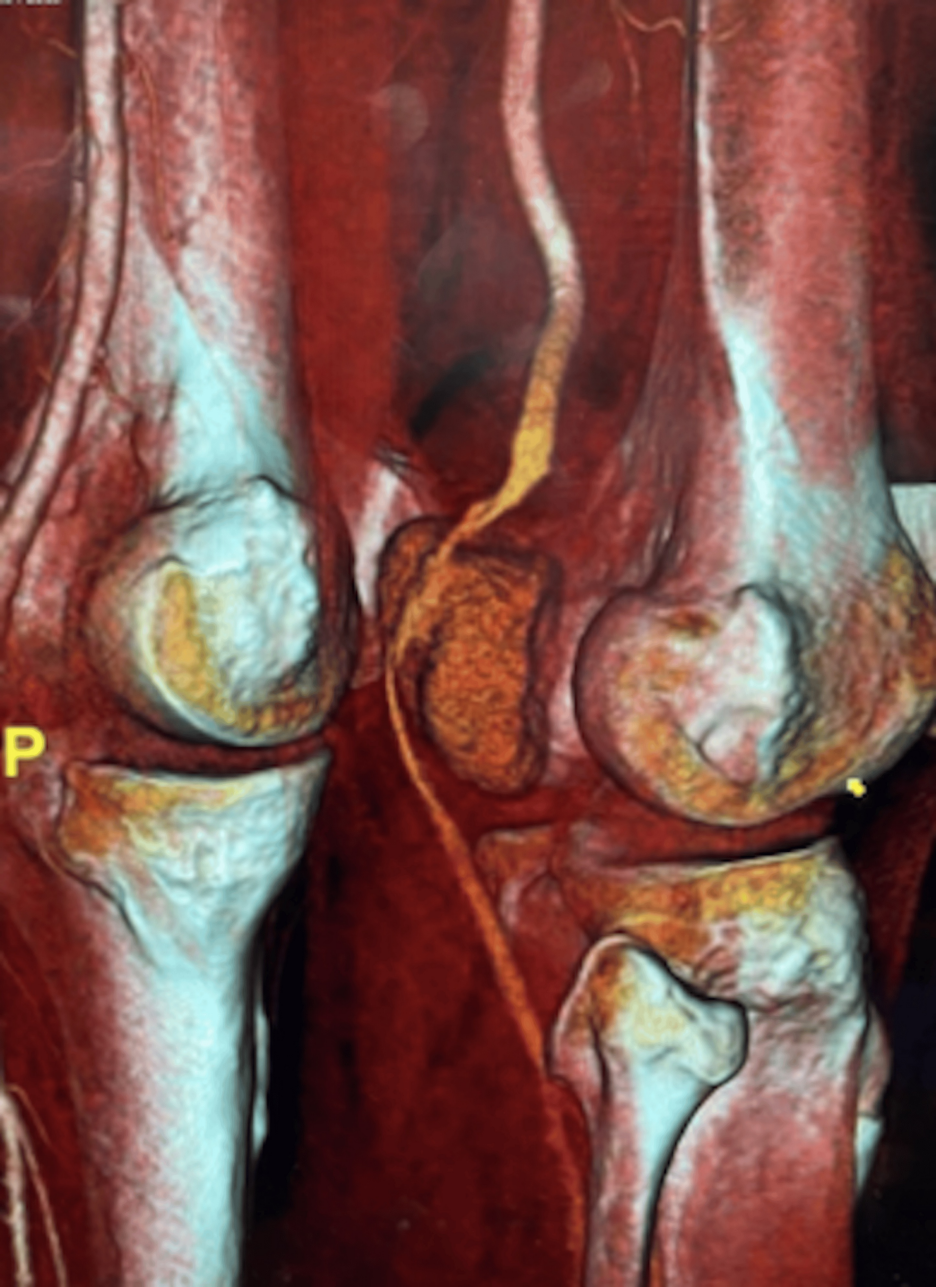
3D reconstruction of preoperative CT angiogram showing posteroateral view of the right popliteal artery

**Figure 2:**
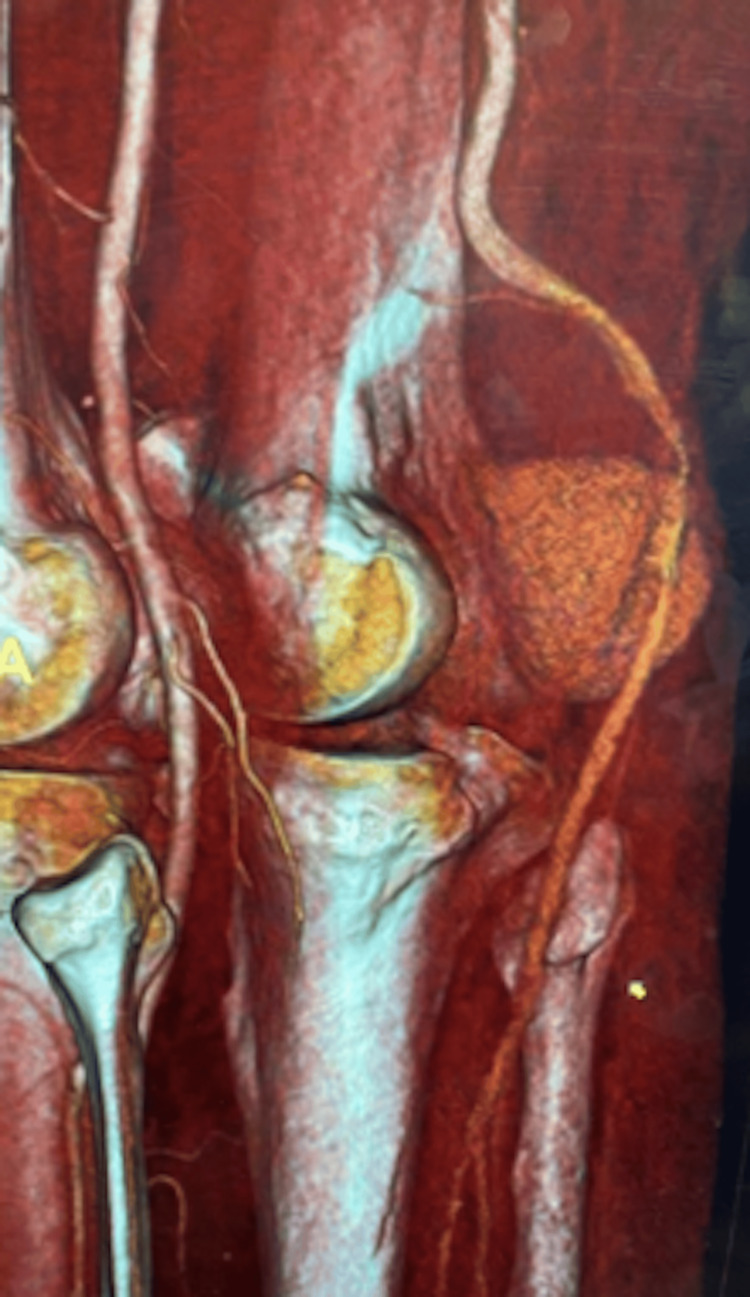
3D reconstruction of preoperative CT angiogram showing posteromedial view of the right popliteal artery

On examination, his peripheral pulses were all present and abdominal examination was unremarkable. Of note, there was an approximately 10 X 10 cm non-tender pulsatile swelling in the back of his right knee joint slightly lateral in his popliteal fossa. The overlying skin was normal and there were no other changes in his leg. His neurological and musculoskeletal exam was otherwise normal. The provisional diagnosis was a popliteal artery aneurysm. Evaluation of this patient involved a CT angiogram of the lower limbs and blood investigations. The CT angiogram was reported as a right popliteal aneurysm of 8.5 cm diameter with a large amount of intramural thrombus (Figures 1, 2).

A discussion in our vascular surgery multi-disciplinary team meeting occurred, where a diagnosis of popliteal artery pseudoaneurysm was made and open surgery was planned.

This diagnosis, was arrived upon due to the large size of the aneurysm and that the swelling was not fusiform. The aneurysm appeared to originate from the anteromedial aspect of the middle part of the popliteal artery, with an approximately 2 cm neck.

There was a difference of opinion regarding the best approach to the repair. The conventional popliteal aneurysmal repair involved an approach from the medial side, bypass the aneurysm using an ipsilateral great saphenous vein and ligation of both proximal and distal ends of the aneurysmal popliteal artery. Given the unusual findings, slightly more lateral location, and a larger size, a more extensive plan along with a back-up plan was made. It was decided to dissect the popliteal artery from a posterior approach and bypass the aneurysm using a vein graft.

Before we could proceed with surgery, the patient received a preoperative routine SARS-Cov-2 test, which came back positive. He was asymptomatic. After consultation with infectious diseases and anesthetics, it was decided to proceed with surgery. 

**Figure 3:**
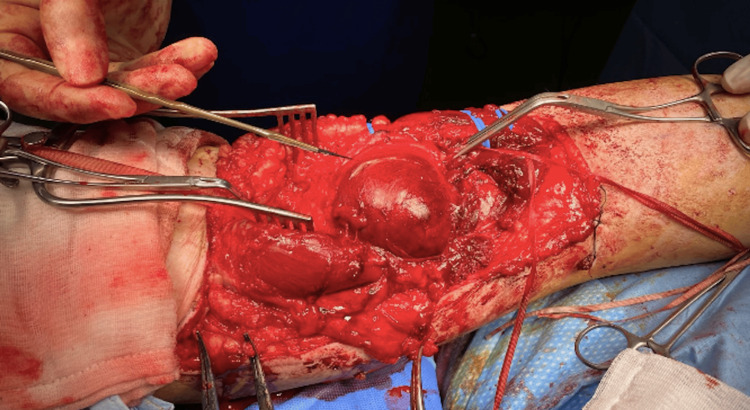
Popliteal artery pseudoaneurysm, fully exposed

A "lazy S" incision was made to approach the nonaneurysmal area above the popliteal artery as it exited the adductor canal. Arterial vascular control was obtained by using vessel loops. Careful dissection was done distally to preserve the sural and tibial nerves. The normal below the knee popliteal artery was dissected out and looped with vessel loops. Gentle dissection was done around the pseudoaneurysm to identify surrounding structures, as seen clearly in Figure 3.

**Figure 4:**
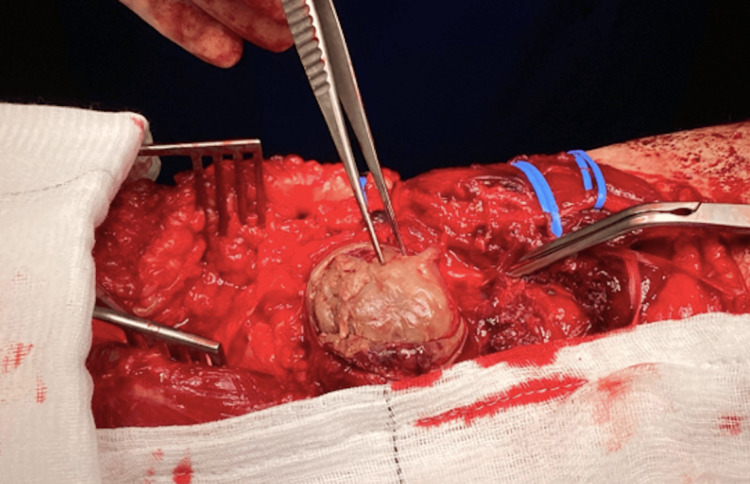
Pseudoaneurysm sac opened and thrombus evacuated

After administration of 5,000 IU of unfractionated heparin intravenously, popliteal artery segments, above and below the aneurysm, were clamped, the pseudoaneurysm was opened, and thrombus was evacuated (Figure 4).

**Figure 5:**
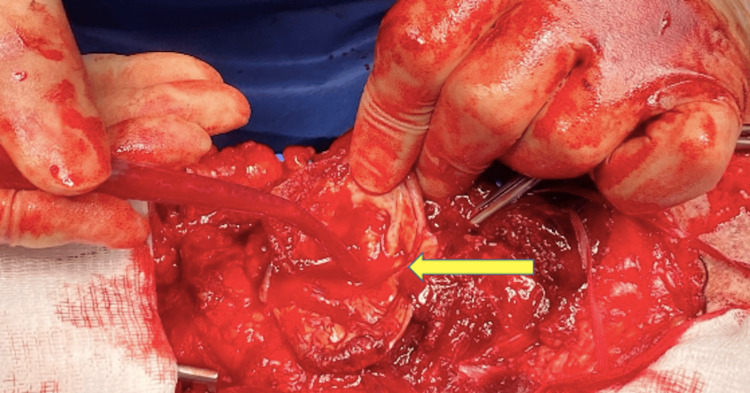
Pseudoaneurysm sac opened and defect seen in the popliteal artery (yellow arrow)

A defect was identified in the anteromedial wall of the popliteal artery about 2 cm in length, as seen in Figure 5, while the rest of the artery appeared healthy.

**Figure 6:**
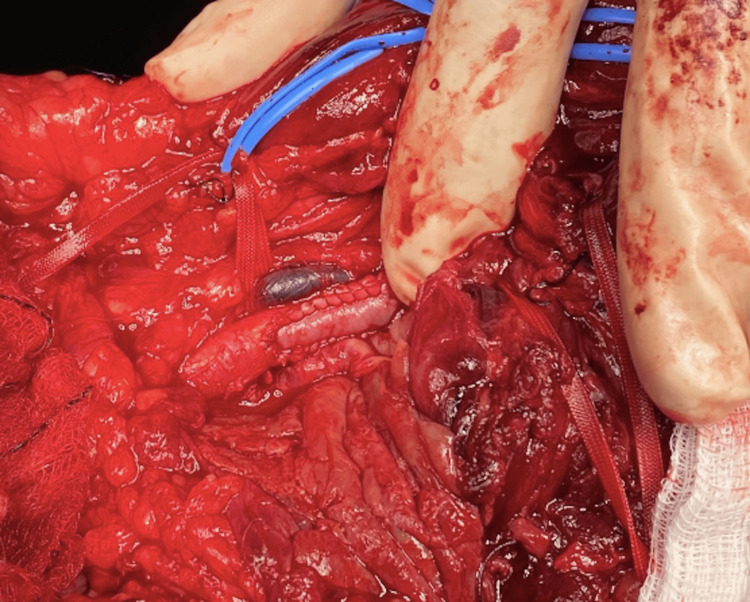
Bovine pericardial patch repair of the popliteal artery defect

The noted defect was extended to examine the intima, which was found to be healthy with no irregularity. After an on-table discussion among the operating surgeons, it was decided to abandon the original plan of a bypass and repair the defect using a biological, bovine pericardial patch, as seen in Figure 6.

The bovine pericardial patch was stitched in place with polypropylene 6-0 sutures. Flow was ensured by using Doppler. Hemostasis was ensured and the pseudoaneurysmal sac was plicated and closed over the patch repair. A 1/8th inch suction passive suction drain was placed in the popliteal fossa and the wound was closed in layers without tension. A partial backslab was applied to prevent the knee from bending, which could affect the repair.

Postoperatively, the patient was well from a surgical point of view and had recovered from COVID-19. He did develop mild symptoms of the COVID-19 infection, but this did not impact on his overall recovery. The 14-day COVID-19 quarantine delayed rehabilitation, but once out of quarantine, he was able to make a swift recovery with physiotherapy intervention. He has regained full range of movement of his knee joint again with no neurovascular deficits.

## Discussion

Our case may have stemmed from a nonideal aspiration of a Baker’s cyst. Imaging may provide important information pertaining to preaspiration, in light of the patient's having an unknown diagnosis of a popliteal swelling [2]. Presumably, the needle penetrated the wall of the artery, which led to extravasation around the artery, much like the case of Kao and Chang [4]. 

Furthermore in a clinical setting, if the knee undergoes any intervention in case swelling occurs post procedure, one should be suspicious of underlying pathology [5]. Ideally, a CT angiogram of the lower limb should be sought to rule out an iatrogenic pseudoaneurysm, but where availability is limited, ultrasound can play a key role [5]. This should be done promptly given the rare risk of a lower limb, developing compartment syndrome as a result of iatrogenic pseudoaneurysm [5].

We treated the patient promptly because of the likelihood of a potential rupture of the pseudoaneurysm with the potential loss of a limb [6]. The open surgery with primary repair, vein patch, and interpositional bypass grafting has been reported as the gold standard for management of this vascular injury; however post-surgical stents may be favorable in orthopedic cases in the future [6]. In looking at available treatment options for the pseudoaneurysm, endovascular repair appears a reasonable alternative to open repair in selected patients with noted good clinical outcomes; however, single vessel run-off disease should be seen as a contraindication to this procedure given the numerous graft failures in this cohort of patients [7].

## Conclusions

In cases of iatrogenic popliteal pseudoaneurysm, a high index of suspicion and prompt vascular imaging are essential. While open surgical repair remains the mainstay, the approach should be tailored to anatomical findings. In this case, primary repair with a bovine pericardial patch proved effective, offering an alternative to venous bypass when arterial damage is limited. Image guidance during cyst aspiration may reduce vascular injury risk. Multidisciplinary evaluation and intraoperative flexibility are key to optimal outcomes.
